# Artificial Intelligence Models in Health Information Exchange: A Systematic Review of Clinical Implications

**DOI:** 10.3390/healthcare11182584

**Published:** 2023-09-19

**Authors:** Sahar Borna, Michael J. Maniaci, Clifton R. Haider, Karla C. Maita, Ricardo A. Torres-Guzman, Francisco R. Avila, Julianne J. Lunde, Jordan D. Coffey, Bart M. Demaerschalk, Antonio J. Forte

**Affiliations:** 1 Division of Plastic Surgery, Mayo Clinic, Jacksonville, FL 32224, USA; 2 Division of Hospital Internal Medicine, Mayo Clinic, Jacksonville, FL 32224, USA; 3 Department of Physiology and Biomedical Engineering, Mayo Clinic, Rochester, MN 55902, USA; 4 Center for Digital Health, Mayo Clinic, Rochester, MN 55902, USA; 5 Department of Neurology, Mayo Clinic College of Medicine and Science, Phoenix, AZ 85054, USA

**Keywords:** artificial intelligence, electronic health record, health information exchange, machine learning

## Abstract

Electronic health record (EHR) systems collate patient data, and the integration and standardization of documents through Health Information Exchange (HIE) play a pivotal role in refining patient management. Although the clinical implications of AI in EHR systems have been extensively analyzed, its application in HIE as a crucial source of patient data is less explored. Addressing this gap, our systematic review delves into utilizing AI models in HIE, gauging their predictive prowess and potential limitations. Employing databases such as Scopus, CINAHL, Google Scholar, PubMed/Medline, and Web of Science and adhering to the PRISMA guidelines, we unearthed 1021 publications. Of these, 11 were shortlisted for the final analysis. A noticeable preference for machine learning models in prognosticating clinical results, notably in oncology and cardiac failures, was evident. The metrics displayed AUC values ranging between 61% and 99.91%. Sensitivity metrics spanned from 12% to 96.50%, specificity from 76.30% to 98.80%, positive predictive values varied from 83.70% to 94.10%, and negative predictive values between 94.10% and 99.10%. Despite variations in specific metrics, AI models drawing on HIE data unfailingly showcased commendable predictive proficiency in clinical verdicts, emphasizing the transformative potential of melding AI with HIE. However, variations in sensitivity highlight underlying challenges. As healthcare’s path becomes more enmeshed with AI, a well-rounded, enlightened approach is pivotal to guarantee the delivery of trustworthy and effective AI-augmented healthcare solutions.

## 1. Introduction

### 1.1. Background

Thanks to the accessibility of electronic patient data, precision in medicine has seen rapid advancements. The Electronic Health Record (EHR) systems not only store patient biographical data but also amass all information gathered from institutions, encompassing radiographic imaging, blood tests, and other diagnostic tests. Consequently, the sheer volume of data, which originates from diverse patient details across various clinics, hospitals, and private care, along with modalities such as imaging, prescriptions, and procedures obtained at disparate times, forms a vast dataset that is notable in its scope and complexity [1,2,3,4].

EHRs are being rapidly adopted by healthcare groups worldwide to improve efficiency and efficacy and reduce care costs [5,6]—47% of nations now use national EHRs [7,8]. 

The Institute of Electrical and Electronics Engineers (IEEE) defines interoperability as the “ability of two or more systems or components to exchange information and to use the information that has been exchanged” [9]. However, since EHR data is often captured in multiple formats, achieving interoperability becomes a significant challenge, given the multitude of unique EHR formats to contend with. Health Information Exchanges (HIEs) can be an answer for fragmented healthcare systems by gathering EHR data from different provider groups and networks into a single, interoperable repository [7,10]. 

HIE is a valuable tool for disease monitoring due to its extensive regional and demographic spread [7,11]. However, merging data from various sources into an HIE could result in possible quality problems, such as dropout, or aggravate the problems present in each EHR system, such as low data integrity [11,12,13,14].

For the interchange, integration, and access of electronic health information, Health Level 7 (HL7) standards such as HL7 v2, HL7 v3, and Fast Healthcare Interoperability Resources (FHIR) specify the vocabulary, structure, and data formats necessary for interaction between systems. These guidelines support professional practice and viewpoints on administering, providing, and assessing healthcare services [5,15,16]. In healthcare, data analytics is segmented into three tiers: predictive, prescriptive, and descriptive. These analytics are crucial in guiding decision-making and enhancing patient outcomes [17].

The healthcare industry is transforming, with advances in Artificial Intelligence (AI), Machine Learning (ML), Natural Language Processing (NLP), and Deep Learning Neural Networks (DNN) reshaping everything from diagnosis to treatments [15,18]. Using FHIR-based algorithms for data analytics can improve health outcomes for acute and chronic conditions while reducing the skill demands within the healthcare system [17,19,20]. However, adopting AI models could change the landscape by potentially increasing healthcare productivity, reducing costs, and minimizing energy consumption [21,22,23,24]. 

### 1.2. Problem Statement and Research Questions

Given the increasing relevance of HIE in the healthcare landscape and the transformative potential of AI, understanding its current applications and limitations within HIE is crucial. Although there has been significant research on the application of AI algorithms with EHRs for different levels of data analytics and decision-making [25,26], a noticeable gap exists in the literature—a comprehensive, systematic review and analysis of AI models specifically within HIE contexts. Our systematic review aimed to answer the following questions:

1. What are the implementations of AI models in the HIE?
2. What is the effectiveness of different AI models in improving clinical outcomes based on HIE data?
3. What are the barriers to the implementation of AI in HIE?
4. What limitations have been identified in current studies, and what potential future research directions are suggested?

### 1.3. Objectives

To answer these questions, we will identify and compile the most recent research on the application of AI in HIE settings. First, we assess the effectiveness of AI applications in monitoring and predicting various pathologies and determine the obstacles in this way. Then, we suggest possible solutions and provide topics for additional study in this field.

## 2. Methods

### 2.1. Search Strategy

A search strategy was implemented aiming to maximize precision and accuracy of yield.
**Research Units****Keywords/Terms**Health Information Systems/Standards“Health Information Exchange”, “Health Level Seven”, “HL7”, “HIE”, “FHIR”, “HIO”, “Fast Healthcare Interoperability Resource”Artificial Intelligence & Data Analytics Methods“Machine Learning”, “Natural Language Processing”, “Artificial Intelligence”, “Logistic Models”

Keywords/terms from each research unit were combined using Boolean operators (AND, OR).

The final search string used was: (“Health Information Exchange” OR “Health Level Seven” OR “HL7” OR “HIE” OR “FHIR” OR “HIO” OR “Fast Healthcare Interoperability Resource”) AND (“Machine Learning” OR “Natural Language Processing” OR “Artificial Intelligence” OR “Logistic Models”).

This search string was formulated based on similar reviews and was utilized to execute the literature search.

### 2.2. Data Sources and Databases Searched

Two independent investigators searched five large digital bibliographic database sources: Scopus, Cumulative Index of Nursing and Allied Health Literature (CINAHL), Google Scholar, MEDLINE (PubMed), and Web of Science to cover the relevant studies adequately. In Scopus, we used the ‘Article Title, Abstract, Keywords’ search field option. Filters were applied to retrieve peer-reviewed journal articles.

In CINAHL, the search was executed in the ‘TX All Text’ field. Filters for ‘Peer-Reviewed’ and ‘Journal Article’ were applied. Google Scholar was searched using the basic search bar. Given the vast number of results from Google Scholar, only the first 100 papers of the search yield were evaluated. In MEDLINE (PubMed), the search was applied to the ‘Title/Abstract’ fields. We used the ‘Journal Article’ filter to refine our results.

The ‘Topic’ field was used to conduct the search at Web of Science, and the ‘Articles’ filter was applied to refine our results.

Our search commenced on 20 March 2023; notably, we did not restrict the search period. The specific features or nuances of each database (e.g., using MeSH terms in PubMed) were considered while formulating the search strings. Any adjustments made to the search strings for each database due to database-specific functionalities were noted.

### 2.3. Study Eligibility and Selection Process 

Inclusion criteria: original research articles describing the use of any form of AI in healthcare management situations with clinically based approaches on the HIE or EHR + HIE dataset. Exclusion criteria: articles that were published in non-peer-reviewed journals, articles in languages other than English, meta-analyses, systematic reviews, literature reviews, preprint studies, commentaries, opinion pieces, pilot studies, prototypes, technical designs, and secondary data analyses, and articles focused solely on data transfer security, HER data, dental/pharmacological procedures, or articles without full-text access. 

**Figure 1:**
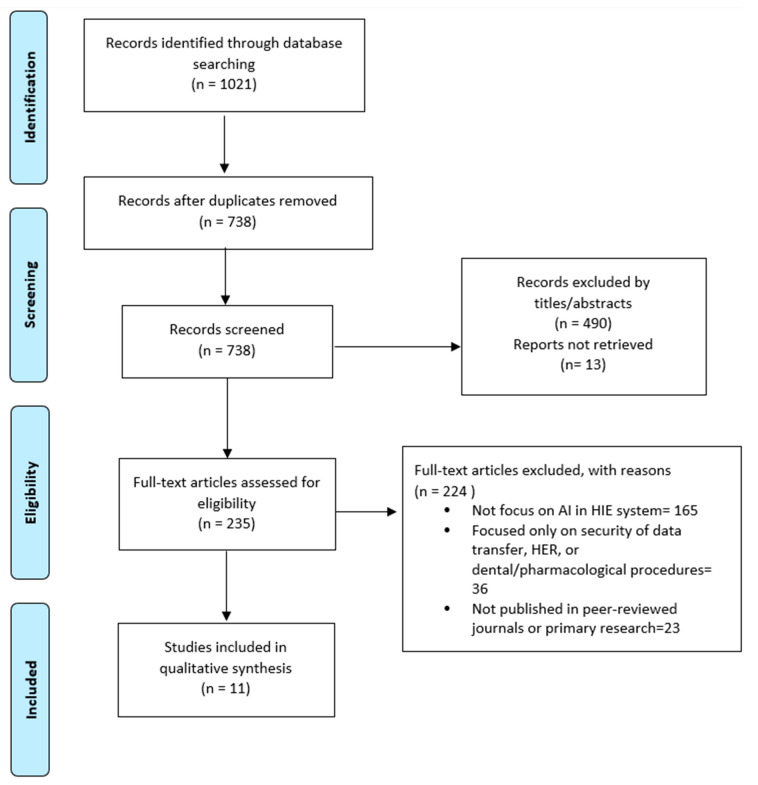
PRISMA flow diagram—study selection process.

We used the Preferred Reporting Items for Systematic Reviews and Meta-Analyses (PRISMA) 2020 statement as the basis of our organization [27] (Figure 1).

After performing the search, papers from each database mentioned above were selected based on the given search string. Next, each report was carefully reviewed chronologically, covering the title, abstract, keywords, introduction, background, methodologies, findings, discussion, and conclusion to ensure thoroughness. Finally, articles were retrieved from the databases if the search phrase or a substring met any article components.

Afterward, we eliminated duplicate articles obtained from different databases and filtered the collected papers using Endnote software (Version 20.4.1.).

### 2.4. Data Quality and Risk of Bias Assessment

We used a three-step method to assess the quality of the chosen articles. First, we carefully considered each article’s title and abstract to ascertain its applicability to our research concerns. Second, we rapidly skimmed the complete piece to ensure it had all the pertinent information. Finally, we reviewed the full paper from start to finish to ensure it was valuable and could answer our research questions. 

The first two authors independently evaluated each study for bias using the QUADAS-2 tool from the Cochrane Library for the quality assessment of diagnostic accuracy studies [28]. Subsequently, a summary and a graph were created using RevMan 5.4 (Cochrane Collaboration), enabling the stratification of bias in diverse areas. Any conflicts among the first two authors were solved by the decision of the third author independently. 

### 2.5. Data Synthesis and Analysis

We collected various data types from each article, including author names, publication years, and study design. We also recorded the answers to our research questions from these articles for further descriptive analysis. 

## 3. Results

### 3.1. Characteristics of Included Studies

#### 3.1.1. Number of Included Studies

**Figure 2:**
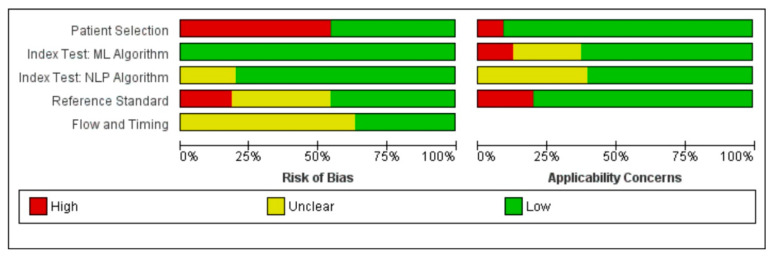
Risk of bias and applicability concerns graph: review authors’ judgments about each domain presented as percentages across included studies. Red stands for high risk of bias, yellow stands for unclear risk, and green stands for low risk of bias.

**Figure 3:**
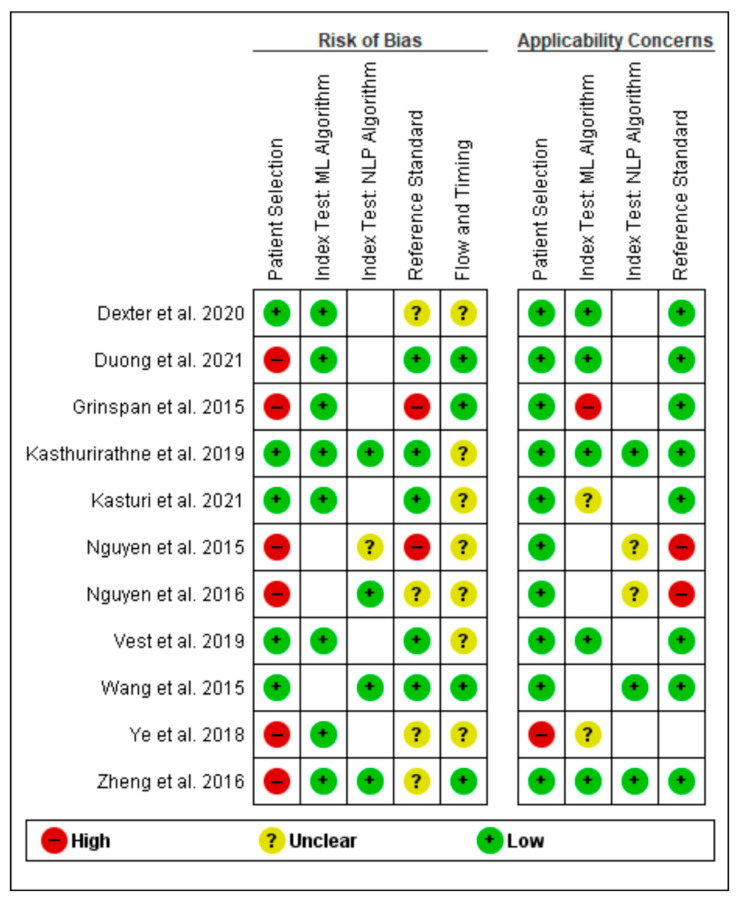
Risk of bias and applicability concerns summary: review authors’ judgments about each domain for each included study. Red stands for high risk of bias, yellow stands for unclear risk, and green stands for low risk of bias [29,30,31,32,33,34,35,36,37,38,39].

Our original search yielded 1021 articles for this systematic evaluation. After the application of eligibility criteria, the result was 11 papers that discussed the application of artificial intelligence models and algorithms in health information exchange and the interoperability of electronic health records. Table 1 shows a bias assessment summary, and a graph of included studies can be seen in Figure 2 and Figure 3.

#### 3.1.2. Type of Studies

Overall, eleven articles were reviewed, encompassing various designs. Specifically, there were seven cohort studies [29,30,33,34,36,38,39], of which three were retrospective [29,36,39]. In addition, there were three retrospective observational studies [32,35,37] and one study that focused on developing and evaluating a model [31].

#### 3.1.3. Parameters Obtained

Type of parameters obtained from the patient’s records were demographics [29,34,35,36,39], risk factors [30,33,34,35], radiology encounters [29,34], histology and cytology reports [31,32], laboratory test results [34,37,38], visit history (ED, outpatient and, inpatient) [29,30,35,36,39], medications [33,34,35,38,39], comorbidities [29,30,35,39], and social determinants of health [35,38,39].

### 3.2. Types of HIE Standard, FHIR Medical Coding Systems, and Application Programming Interface (API)

The type of HIE used in the studies was Health Level 7 (HL7) [31,32,37,38]. Other studies used the specific HIE resources without mentioning the standard, such as the NYCLIX HIE network [29], Indiana Network for Patient Care (INPC) [35,36,39], and Maine Health Information Exchange (HealthInfoNet, HIN, Mount Lawley, WA, USA) [30,33,34].

The HIE medical coding systems used for data extraction were International Classification of Diseases (ICD)-9 codes [29,36,38], ICD-9-CM codes [30,33,34], ICD-10 codes [36], ICD-10-CM codes [34,38], Systematized Nomenclature of Medicine—Clinical Terms (SNOMED CT) [30,31,32,33], Logical Observation Identifiers Names and Codes (LOINC) codes [37] and ICD-O. Additionally, two studies did not mention the coding system they used [35,39].

Application Programming Interface (API), such as Java Messaging Service (JMS) API, was implemented in one of the included studies [31].

### 3.3. Types of AI Models, Applicability, and Validation

Most of the studies used ML models as the basis of their work (N = 8) [29,33,34,35,36,37,38,39], out of which two studies used both ML and NLP algorithms [33,36]. Three studies used NLP as their only prediction model [30,31,32]. At the same time, the most used NLP model was Medtex [31,32]. 

Meanwhile, five studies applied the Random Forest algorithm to train their AI model; however, three studies used different types of decision trees other than random forest [29,33,35].

The AI models included in this review were applied for different purposes—seven authors used the models as a prediction tool [29,34,35,36,37,38,39]; however, five used them to help with data extraction [30,31,32,33,36,39], and two for case finding [30,33]. Of these studies, cancer [31,32] and heart failure [30,38] were the most studied outcomes.

Several methods were implemented to validate and evaluate the effectiveness of the AI model applicability, including the holdout validation method (N = 4) [29,36,37,39], cross-validation method (N = 2) [30,35], prospective cohort (N = 4) [30,33,34,38], manual chart-review (N = 4) [30,31,32,33], and statistical methods (N = 1) [30]. 

### 3.4. Models Metric Scores

Overall, the studies were able to predict the frequency of emergency department (ED) visits. At the same time, some of them focused on people with epilepsy [29] with AUCs ranging from 0.78 to 0.88, indicating very good predictability, fair to good PPV (60–81%), and calibration (5–15%). However, sensitivity was uniformly poor (12–30%). Vest et al. [35] studied revisit rates over set periods and developed ML and NLP models from five distinct datasets. One model, based on census travel social determinants, had a 61% AUC. Another using patient-level EHR data from the current visit had 69.6% AUC. A third model, using historical patient EHR data, achieved 70.7% AUC. They also developed a model from HIE data with 71.3% AUC and one combining current and past visits, which reached 73.2% AUC.

Reference [36] utilized random forest and NLP to identify depression patients and predict their advanced care needs, achieving AUC scores of 86.31–94.43% for high-risk groups and 78.87% overall. Meanwhile, 8.29% needed advanced care.

An automated system for cancer registry alerts from Medtex showed promising results: F1 scores ranged from 89.6% to 96.5%, and it categorized cancer traits with a recall of 0.78 and a precision of 0.83 [31,32].

Free-text laboratory data were used to train AI algorithms to identify three notifiable diseases: salmonella, histoplasmosis, and syphilis [37]. The ROC-AUC was 99.22%, 99.91%, and 99.18% for syphilis, salmonella, and histoplasmosis, respectively.

Duong et al. [38] evaluated a predictive model to detect heart failure. The model showed an AUC of 82.4%, while the Wang et al. [30] case-finding algorithm achieved 69% sensitivity, 98.8% specificity, and 78.9% F-measure. However, [39] utilized an ML model to predict healthcare resource use among COVID-19 patient subgroups. The AUC-ROC for the first week was 88.71% and 86.21% for the initial six weeks. Corresponding F1 scores were 61.81% and 61.36%, respectively.

On the other hand, Zheng et al. [33] looked into creating a decision tree-based model to detect the presence of diabetes mellitus in patients. In retrospective blind testing using an NLP-based algorithm, the model obtained 62% sensitivity and 99.4% specificity, while prospective verification yielded 68% sensitivity and 98.5% specificity.

Using prior-year medical data, Chengyin Ye et al. devised an algorithm predicting hypertension risk for the next year. Their XGBoost model achieved AUCs of 91.7% retrospectively and 87% prospectively. [34]

## 4. Discussion

### 4.1. Implications and Key Findings

According to this systematic review, using AI models in HIE may have potential benefits. First, it may enhance the precision and effectiveness of patient management based on HIE. These AI models and algorithms may accomplish various tasks, such as data extraction, clinical decision assistance, and prognosis prediction. In addition, AI may forecast multiple health-related results, such as cancer, sepsis, heart failure, in-hospital cardiac arrest, and COVID-19-related resource utilization [30,32,40,41,42,43]. Several measures, including area under the curve (AUC), precision score, positive predictive value (PPV), negative predictive value (NPV), sensitivity, specificity, calibration, and F-measure, were used to evaluate the performance of algorithms. In a clinical context, a higher AUC indicates better reliability in distinguishing between patients with and without the condition. The study’s AUC of 90% (ranging from 61% to 99.91% across studies) demonstrates the model’s strong ability to differentiate between these patient groups. Sensitivities nearing 96.5% (range: 12% to 96.5%) and a recall of 78% both highlight effective detection of true positives—critical for accurate diagnosis. Meanwhile, a specificity of 98.80% (range: 76.30% to 98.80%) limits false positives, reducing unneeded interventions. The model’s positive and negative predictive values, 83.70% and 94.10%, respectively, underscore their accuracy in both confirming and ruling out the condition. A precision rate of up to 88% guarantees most positive detections are correct, refining treatment approaches. The harmony between the model’s precision and recall is further shown by an F-measure that reaches 96%, and the limited classification error range (5.17% to 5.67%) supports the algorithm’s consistent accuracy in clinical applications.

#### 4.1.1. Health Information Exchange, Fast Healthcare Interoperability Resources, and Application Programming Interface

The “digital health” area is expanding quickly and uses digital tools to enhance population health, patient outcomes, and healthcare administration [44,45,46,47,48].

Although access to patient information for clinical treatment is the primary purpose of HIEs, data gathered by HIEs may also serve a secondary purpose in public health by helping to track disease and estimate its burden at the community level [7,10,49,50]. 

Efforts at uniformity in the HIE area include using coding systems and medical thesauruses. These are employed to categorize medical data and avoid repetition and misunderstanding in medical terminology [51,52,53,54]. FHIR is the most current HL7 system standard [54]. It was first introduced in March 2014, and multiple technical design studies conducted between 2018 and 2022 favored FHIR as their preferred standard [40,41,42,43]. HL7 messaging systems were used by [31,32,37,38] to gather their input information, and some authors tried to improve their data collection quality using HL7 version 2. Nevertheless, adopting the most recent standard, FHIR can increase the study’s reliability due to its modern design, integrated data exchange, standardized resources, and enhanced support for current healthcare use cases, such as patient portals [41,55,56,57]. However, other studies conducted during different time frames did not specify the standard used [29,30,33,34,35,36,39].

Web services are created using a collection of architectural concepts called REST (Representational State Transfer). Using pre-existing web standards such as RESTful Application Programming Interfaces (API) and XML or JSON data exchange formats, which are lightweight and easy for individuals and machines to understand, has helped FHIR gain preference [5,58]. For HIEs, using Restful API offers many advantages, including scalability, speed, and adaptability, as demonstrated by Amrollahi et al. [42], Tseng et al. [41], and Henry et al. [43]. Still, it is also necessary to handle their complexities and security risks. Java Messaging Service (JMS) API is another reliable and scalable messaging system [59] interface that was used by Nguyen et al. [31].

The research emphasizes how crucial standardization is to health HIE networks. In particular, HL7 was widely utilized in the studies examined, which implies that policymakers and healthcare organizations should prioritize the adoption and implementation of this system, specifically FHIR, to ensure effective and efficient data sharing across various healthcare systems.

#### 4.1.2. FHIR Medical Coding System

Globally, disorders and health conditions are categorized using the International Classification of Diseases (ICD) for medical documents and mortality certificates [5,60,61]. FHIR also makes use of a variety of system identifiers. For example, the Logical Observation Identifiers Names and Codes (LOINC) system distinguishes clinical and laboratory data such as blood tests, vital signs, and medical histories. Henry et al. [43] and Dexter et al. [37] used LOINC to obtain observation data from the FHIR server and handle their automated laboratory reports, respectively.

Systematized Nomenclature of Medicine—Clinical Terms (SNOMED CT), a specific and international clinical terminology used to define clinical concepts such as illnesses, treatments, and medications, is a different coding system [5,58,61,62]. Out of 11, 7 studies used various versions of ICD coding systems, such as ICD-9, ICD-10, and ICD-O [29,30,31,33,34,36,38]. Based on the population and nature of the research, each of these versions can be used depending on its unique characteristics. 

ICD primarily focuses on categorizing illnesses, accidents, and causes of death. However, SNOMED CT offers a more thorough representation of clinical concepts that can be utilized, for instance, to map spans in pathology reports to clinical concepts [31,32] or to develop a controlled set of medical terms related to CHF [30].

The significance of using a uniform classification system is vital. For example, using ICD to retrieve information from patient records to guarantee uniformity and precision in data retrieval for AI algorithms is crucial to healthcare systems and must be implemented.

#### 4.1.3. AI Models in Healthcare Data Exchange

AI describes algorithms that can perform duties corresponding to human cognitive abilities such as logic [26]. Improved patient outcomes and increased productivity are the goals of applying ML to patient care [63,64,65,66,67], with some models even surpassing human decision-makers in some situations [68].

AI models have demonstrated enhanced capabilities in managing health-related big datasets. Not only can they process upwards of 250 million images cost-effectively [69], but they also excel in creating “digital twins” by constructing comprehensive data infrastructures that encompass patient treatment histories, outcomes, and physiological parameters [70,71].

**Figure 4:**
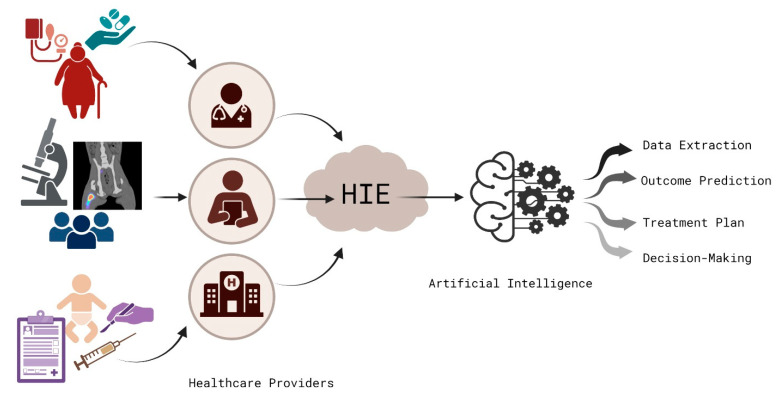
AI implication in Health Information Exchange System.

The two most popular ML models in healthcare are explanatory and predictive, with explanatory frameworks used to evaluate causal theories and predictive models for predicting new data. Predictive models include decision trees and random forests, which employ rule or tree-based reasoning. Although logistic and linear regression models are mainly used for prediction, they can also serve as explanatory models. Conversely, neural networks can be used for prediction and explanation tasks [15,72,73,74,75]—Figure 4 [76].

Despite the high degree of predictability and performance ratings that all AI models demonstrated, there are still some significant constraints. For example, to predict the incidence of emergency department visits by individuals who have epilepsy, Grinspan et al. [29] employed ML modeling, but they did not differentiate between visits for epilepsy and visits for other causes or between ED discharges and visits leading to inpatient stays. Inversely, Kasturi et al. addressed this problem by thoroughly examining the patient codes to only consider patients with COVID-19 diagnosis and not the admissions due to other causes such as accidents. Additionally, because the research was performed at four institutions in a single urban region, it may be less accurate to make general predictions using machine learning [29,31,32,34,37,38,39]. 

To overcome this limitation, Nguyen et al. [31] account for deviations frequently found in health data, such as missing data and skew distributions. They evaluated seven different predictive modeling methods in this regard. However, since their system has not been explicitly trained on some hidden categories, it may be more challenging to classify data correctly in this case. The restricted data categories utilized, especially those influencing prognostic capabilities such as insurance details and physician profiles, as highlighted in [29], might also impact the models’ predictive accuracy. 

Using a year of EMR data, researchers predicted a patient’s likelihood of a first-time HF diagnosis using the XGBoost ML model. This model found a group with over nine times the HF risk compared to the baseline. However, it is important to mention that the study’s omission of intervention effects on the predictive model could potentially misguide decision-makers [38]. Reference [30] also attempted to create a case-finding algorithm for detecting individuals with CHF employing only NLP analytics. The algorithm received outstanding results regarding sensitivity, specificity, and F-measure. However, the availability of CHF markers limited their contributions. 

NLP, DNN, and ML models can categorize primary cancer types and forecast cancers of unclear origin [40]. NLP may be the best option for recognizing clinical notes that are not coded. Since clinical notes frequently use informal, unstructured language that may not adhere to a standardized vocabulary or style, it is challenging for conventional rule-based systems to recognize and retrieve information precisely. One of the possible drawbacks is the potential for missing diagnostic codes, which could restrict how broadly the NLP-based approach could be applied [33,77,78,79,80,81]. This is also applicable to [35] even though their ML-based HIE data model displayed a higher AUC in contrast to EHR patient-level data, as there is still a chance that the results will not be generalizable due to a potential lack of data availability in various HIE systems.

Nguyen et al. used the General Architecture For Text Engineering (GATE) platform-based Medtex medical text analysis system to obtain high levels of predictability with a sensitivity level of 96.50%. However, the increased frequency of false positives diminished the study’s PPV [32]. In another study, they used the same Java-based NLP software (Medtex, version GATE 4.0 build 2752) platform to assess the service’s ability to process a large volume of HL7 pathology messages. Again, the results were promising, with an F-measure of 0.80 [31]. 

As reported by [39], their decision tree-based ML model predicted healthcare resource utilization for COVID-19 patients but showed notable discrepancies between different demographic factors such as age, ethnicity, and gender. In addition, each model had lower-than-ideal recall results, suggesting that many individuals needing medical attention were neglected. This emphasizes how critical it is to conduct research with strict population confounding controls to analyze the effects of various factors across models. These discrepancies can have a significant impact, particularly on residents of underserved areas, and they can also contribute to healthcare disparities related to age [82,83,84].

The performed studies had the opportunity to work with a vast amount of data, but the time period for examining their models was generally limited. Most of the studies chose their patient data for a timeline of a year or two [29,30,32,33,37], while [34,38] extended the frame to three years, and only two studies chose their data for more than five years [35,36]. This issue may prevent the data from providing models with a full view of long-term risk, making it challenging to spot trends or forecast results and leading to ineffectual therapy or intervention.

The analyzed studies revealed confident outcomes in identifying diseases such as cancer, heart failure, and HTN. Therefore, to improve the accuracy and efficiency of diagnostics, healthcare organizations should consider incorporating AI models and algorithms into their diagnostic processes.

### 4.2. Strengths and Limitations of the Review

We discussed various AI uses in HIE data, such as data extraction, prediction, and clinical decision support. Additionally, to increase the validity and trustworthiness of our results, we used a strict, transparent approach for identifying, selecting, and evaluating relevant studies. There are some restrictions on the quality and variety of the included research. As a result, it may be challenging to come to a definitive conclusion about the utility and generalizability of the applied AI methods.

Studies with favorable or noteworthy findings may be more likely to be published and included in the review, making them susceptible to bias.

The AUC serves as a pertinent metric for evaluating model performance; however, its comparison across disparate studies demands caution due to variances in context and data intricacies. Notwithstanding the promise that high AUC values indicate in HIE, it remains imperative to rigorously evaluate each model in its intended context to ascertain its appropriateness and efficacy.

This study does not thoroughly examine the ethical, legal, and social aspects of using AI in HIE data, even though they are crucial factors in developing and applying AI systems in healthcare.

### 4.3. Future Directions and Recommendations

1. Future research should utilize diverse data sources (e.g., imaging records and lab findings) and explore alternative machine-learning methods to improve the model’s ability to identify a broader range of disorders.
2. Include diverse patient populations and real-world settings to assess technical design effectiveness and conduct longer-term risk evaluations.
3. To ensure inclusivity, future research should implement strict population confounding controls, critically analyze effects across models, and prioritize addressing healthcare disparities, with a focus on underserved areas and age-related concerns.
4. To optimize model accuracy and predictive value, studies should account for intervention effects, balance sensitivity with positive predictive value, and try to integrate all pertinent predictive markers to enrich model contributions.
5. Further exploration of the ethical, legal, and societal implications of AI in HIE data can enhance our understanding of the significant challenges and issues in healthcare AI.

## 5. Conclusions

In our comprehensive review of 11 distinct research studies, several significant trends emerged regarding the integration of AI with HIE. A clear predilection for ML models was noted in forecasting clinical outcomes, particularly in the domains of cancer and heart failure, pointing to specific areas where AI can make substantial contributions. Additionally, while the HL7 standard has become the benchmark for HIE, the frequent adoption of ICD and SNOMED CT underscores their importance in data retrieval processes. The impressive predictive capacities of the models, as indicated by the AUC metrics spanning from 61% to 99.91%, are however juxtaposed with a wide-ranging sensitivity, highlighting both the potential and challenges of AI in this domain. As we delve into the intricacies of adopting AI in healthcare documentation, it is evident that a more holistic understanding is crucial. The findings underscore not just the complexity but the necessity for meticulous planning, understanding, and continuous exploration to navigate potential pitfalls such as prediction inaccuracies and biases. Only through rigorous analysis from diverse perspectives can we pave the way for practical and reliable AI-driven solutions in healthcare’s future.

## Figures and Tables

**Table 1:** Characteristics of the included studies.

Author and Year	Study Design	Subjects	Outcome	HIE Network	FHIR Coding System	AI Model/Algorithms	Level of Implementation	Validation Method	Metric Score
Grinspan et al., 2015 [29]	Retrospective Cohort	8041 Patients	Epilepsy	NYCLIX, Manhattan, US	ICD-9	ML: LR, Lasso elastic LR, DT, RF, AdaBoost, CART, SVM	Prediction	Holdout	AUC: 78–88% Sensitivity: 12–30% PPV: 60–81% Calibration: 5–15%
Wang et al., 2015 [30]	Cohort	18,295 Patients	CHF	HealthInfoNet, Maine, US	ICD-9-CM SNOMED CT	NLP: RF	Data Extraction Case Finding	Prospective Cohort Manual Review Mann–Whitney Test Cross-Validation	RC: Sensitivity: 69%, Specificity: 98.80%, PPV: 92%, NPV: 94.10%, F1: 78.90% PC: Sensitivity: 64%, Specificity: 98.80%, PPV: 91.40%, NPV: 93.20%, F1: 75.30%
Nguyen et al., 2015 [31]	Development and Evaluation	500 Pathology Reports (201 notifiable cancer)	Notifiable Cancer	QCCAT, Queensland, Australia	SNOMED CT ICD-O	NLP: Medtex	Data Extraction	Manual Review	Recall: 78% Precision: 83% F-measure: 80%
Nguyen et al., 2016 [32]	Retrospective Observational	45.3 M Pathology Reports (119,581 histology and cytology)	Notifiable Cancer	QCCAT, Queensland, Australia	SNOMED CT	NLP: Medtex	Data Extraction	Manual Review	Sensitivity: 96.50% Specificity: 96.50% PPV: 83.70% F1: 89.60%
Zheng et al., 2016 [33]	Cohort	1.12 M patients (retrospective) 935,891 patients (prospective)	DM	HealthInfoNet, Maine, US	ICD-9-CM SNOMED CT	ML: RF NLP: DT	Data Extraction Case Finding	Manual Review Prospective Cohort	RC: Sensitivity 62%, Specificity 99.40%, PPV 95.40%, NPV 92.90% PC: Sensitivity 68%, Specificity 98.50%, PPV 90.10%, NPV 93.90%
Ye et al., 2018 [34]	Cohort	823,627 Patients (retrospective) 680,810 Patients (prospective)	HTN	HealthInfoNet, Maine, US	ICD-9-CM ICD-10-CM	ML: XGBoost	Prediction	Prospective Cohort	RC: AUC 91.70% PC: AUC 87%
Vest et al., 2019 [35]	Retrospective Observational	279,611 Patients	ED Visits	INPC, Indiana, US	N/A	ML: DT	Prediction	Holdout Cross-Validation	Travel SDOH: AUC 61% EHR with current visit data: AUC 69.60% EHR with Prior visit data: AUC of 70.70% HIE data: AUC of 71.30% All data: AUC 73.20%
Kasthurirathne et al., 2019 [36]	Retrospective Cohort	84,317 Patients	Depression Advanced Care	INPC, Indiana, US	ICD-9 ICD-10	ML: RF NLP	Data Extraction Prediction	Holdout	High-risk patients AUC: 86.31–94.43% Overall patient AUC: 78.87% Sensitivity: 68.79–83.91% Specificity:76.03–92.18%
Dexter et al., 2020 [37]	Retrospective Observational	1.7 M Laboratory Reports	Syphilis Salmonella Histoplasmosis	INPC, Indiana, US	LOINC	ML: RF	Data Extraction Prediction	Holdout Laboratory-Level Holdout	Syphilis: AUC: 99.22%, Recall: 91%, Precision: 89%, F1: 90% Salmonella: AUC: 99.91%, Recall: 95%, Precision: 97%, F1: 96% Histoplasmosis: AUC: 99.18%, Recall: 96%, Precision: 88%, F1: 92%
Duong et al., 2021 [38]	Cohort	497,470 Patients (retrospective) 521,347 Patients (prospective)	HF	HealthInfoNet, Maine, US	ICD9 ICD-10-CM	ML: XGBoost	Prediction	Prospective Cohort Cross-Validation	AUC: 82.40% (81.80–83%) Sensitivity: 29.20% Specificity: 97.10% PPV 10% NPV 99.20%
Kasturi et al., 2021 [39]	Retrospective Cohort	96,026 Patients	Health Care Resource Utilization (COVID-19)	CoRDaCo, INPC, Indiana, US	N/A	ML: XGBoost	Prediction	Holdout	1 Week: AUC: 88.74%, Sensitivity: 52.50%, Specificity: 95.78% 6 Weeks: AUC: 86.21%, Sensitivity: 52.57%, Specificity: 94.26%

Abbreviations: CART: Classification and Regression Trees, CHF: Congestive Heart Failure, CPT: Current Procedural Terminology, DM: Diabetes Mellitus, DT: Decision Tree, HTN: Hypertension, ICD: International Classification of Diseases, ICD-9-CM: International Classification of Diseases-9th Revision-Clinical Modification, ICD-10-CM: International Classification of Diseases-10th Revision-Clinical Modification, IHCA: In-Hospital Cardiac Arrest, INPC: Indiana Network for Patient Care, LR: Logistic Regression, LOINC: Logical Observation Identifiers Names and Codes, NLP: Natural Language Processing, NYCLIX: New York Clinical Information Exchange, PC: Prospective Cohort, QCCAT: Queensland Cancer Control Analysis Team, RC: Retrospective Cohort, RF: Random Forest, SDOH: Social Determinants Of Health, SNOMED CT: Systematized Nomenclature of Medicine—Clinical Terms, SVM: Support Vector Machines, XGBoost: eXtreme Gradient Boosting.

## Data Availability

No new data were created or analyzed in this study. Data sharing is not applicable to this article.
